# Supplementary material for: Prediction of harvest-related traits in barley using high-throughput phenotyping data and machine learning
Source: Front Plant Sci. 2025 Oct 14;16:1686506. doi: 10.3389/fpls.2025.1686506 (PMC12560056; doi:10.3389/fpls.2025.1686506)
Supplement: Supplementary Figure 1 — Overview of the experimental timeline and phenotyping protocol. Phenotyping was conducted from the tillering stage to the maturity stage, followed by final harvest for harvest-related traits assessment. The drought stress was induced at the tillering stage, where the pot weight was maintained at 25% soil relative water content (SRWC), and during the flowering stage, the intensity of drought stress increased at 20% SRWC. The phenotyping protocol was conducted daily with different protocols using the PlantScreen™ Modular phenotyping platform at PSI Research Center. In chlorophyll fluorescence imaging using FluorCam (FC), 1FC morning measurement for the quantum yield of PSII (QY_Lss), 2FC night measurement using conditional and high light levels, 3FC morning measurement for chlorophyll content were conducted, thermal infra-red imaging (IR), RGB including two angles from RGB1 side view and one angle RGB2 top view, and hyperspectral imaging (HS) including SWIR and VNIR imaging. And daily weighing and watering (WW) to maintain the target weight. [file DataSheet1.pdf]

SUPPLEMENTARY FIGURES

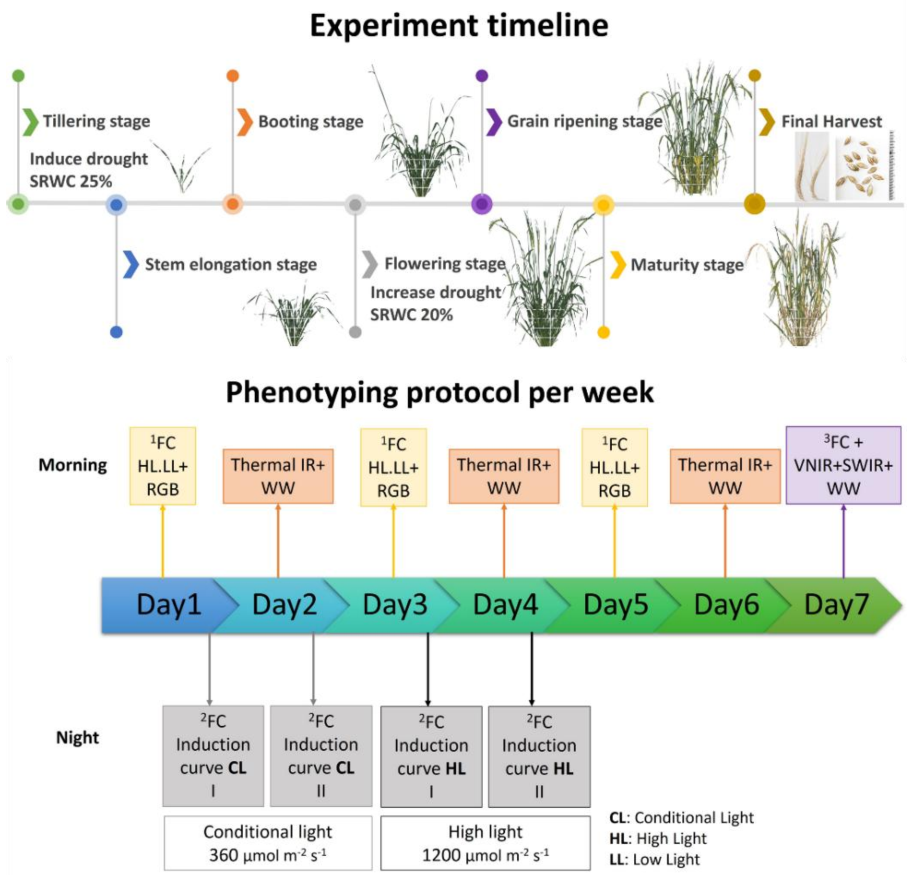

Supplementary Figure S1. Overview of the experimental timeline and phenotyping protocol. Phenotyping was conducted from the tillering stage to the maturity stage, followed by final harvest for harvest-related traits assessment. The drought stress was induced at the tillering stage, where the pot weight was maintained at 25% soil relative water content (SRWC), and during the flowering stage, the intensity of drought stress increased at 20% SRWC. The phenotyping protocol was conducted daily with different protocols using the PlantScreen^{TM} Modular phenotyping platform at PSI Research Center. In chlorophyll fluorescence imaging using FluorCam (FC), ^{1}FC morning measurement for the quantum yield of PSII (QY_Lss), ^{2}FC night measurement using conditional and high light levels, ^{3}FC morning measurement for chlorophyll content were conducted, thermal infra-red imaging (IR), RGB including two angles from RGB1 side view and one angle RGB2 top view, and hyperspectral imaging (HS) including SWIR and VNIR imaging. And daily weighing and watering (WW) to maintain the target weight.

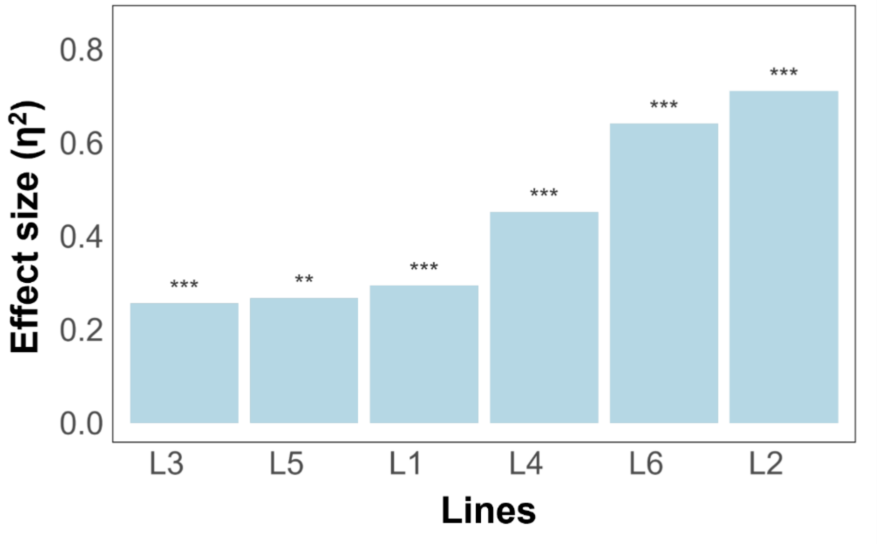

Supplementary Figure S2. Ranking the lines according to their susceptibility to drought stress on harvest traits. PERMANOVA was used to quantify the significance and effect size of the treatment on harvest traits. The asterisks represent significance level P-value < 0.001 for *** and 0.01 for **.

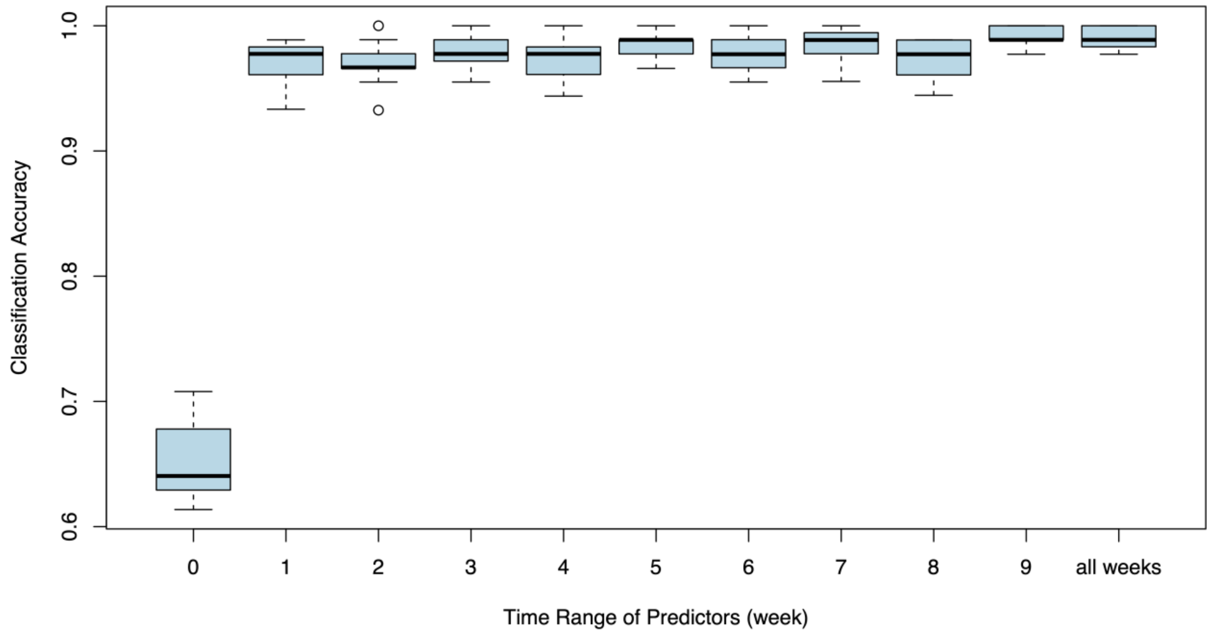

Supplementary Figure S3. Temporal phenomic classification (TPC) of treatment (drought / control) using predictors subset by week. TPC was performed using random forest models. Predictors from each week were used separately along with a model trained on the full data set (all weeks).

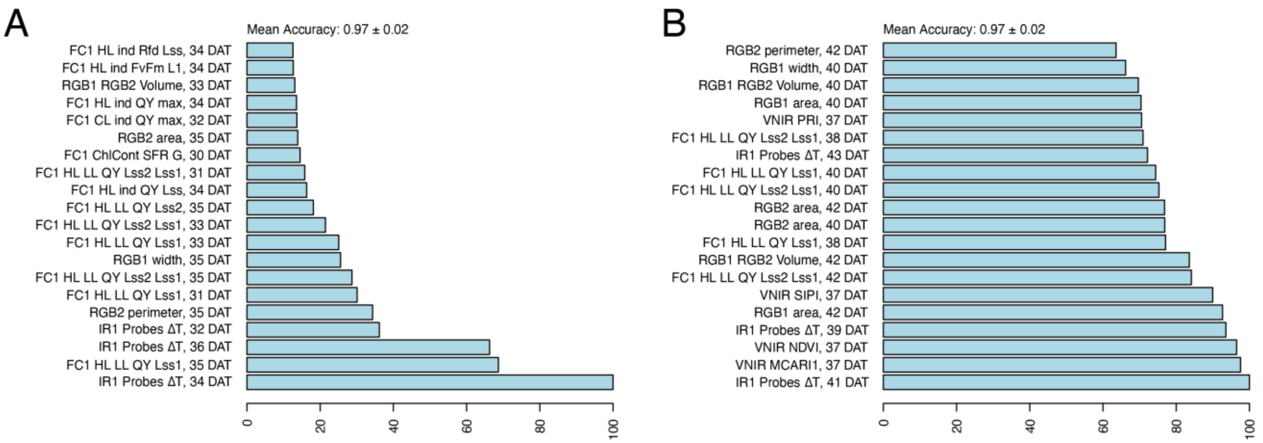

Supplementary Figure S4. Variable importance of temporal phenomic classification (TPC) of treatment using predictors from two weeks. (A) Traits from week 1 and **(B)** week 2 after inducing drought stress. TPC was performed using random forest models.

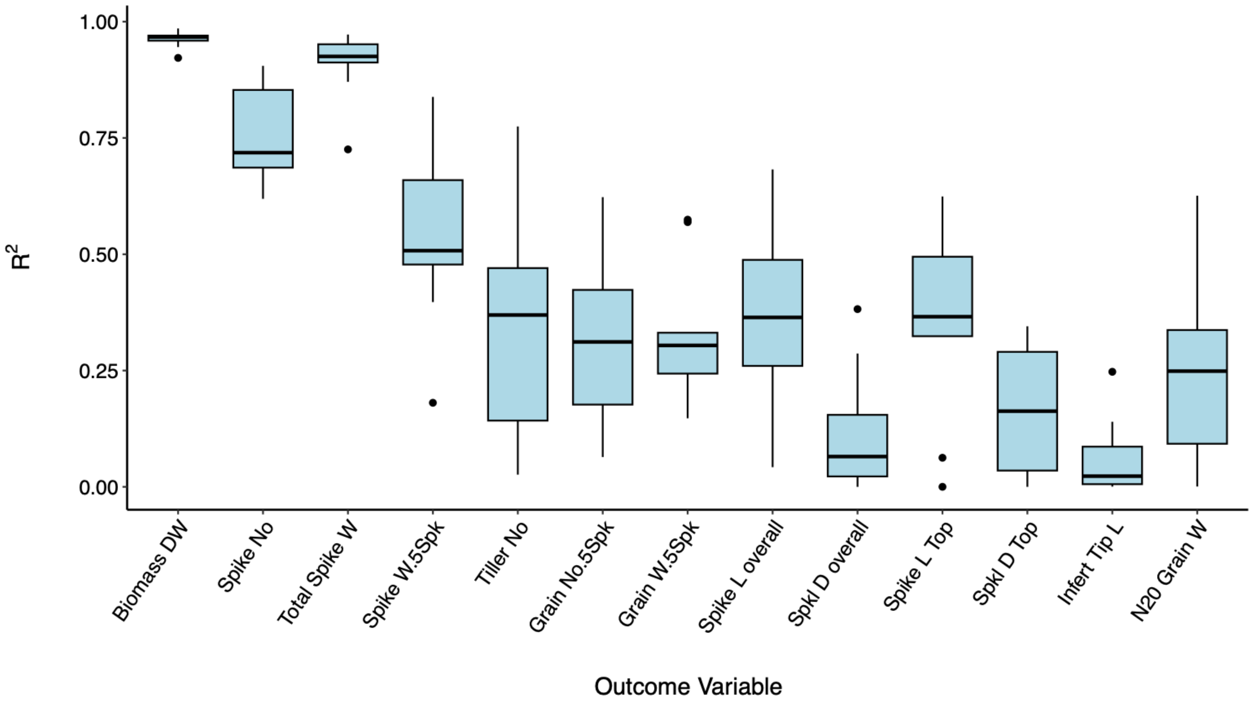

Supplementary Figure S5. Boxplots showing the accuracy (determined by R^2) among all the harvested traits using the LASSO model trained using leave-one-line-out validation instead of the repeated 3-fold CV (9 validations instead of 45).

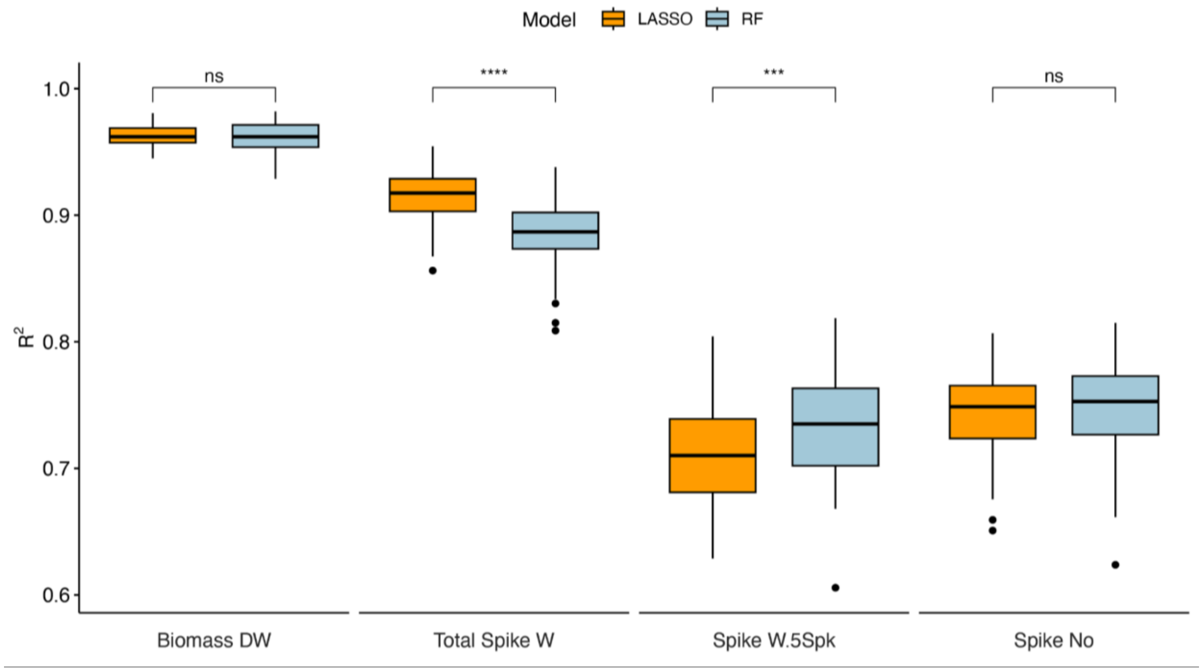

Supplementary Figure S6. Comparison between the accuracy of models. Boxplots showing the accuracy (determined by R^2) among selected harvested traits using LASSO and Random Forest models trained on a pooled non-aggregated dataset. The significance level was determined as *** for P < 0.001, **** for P < 0.0001 and ns for non-significant differences between the models.

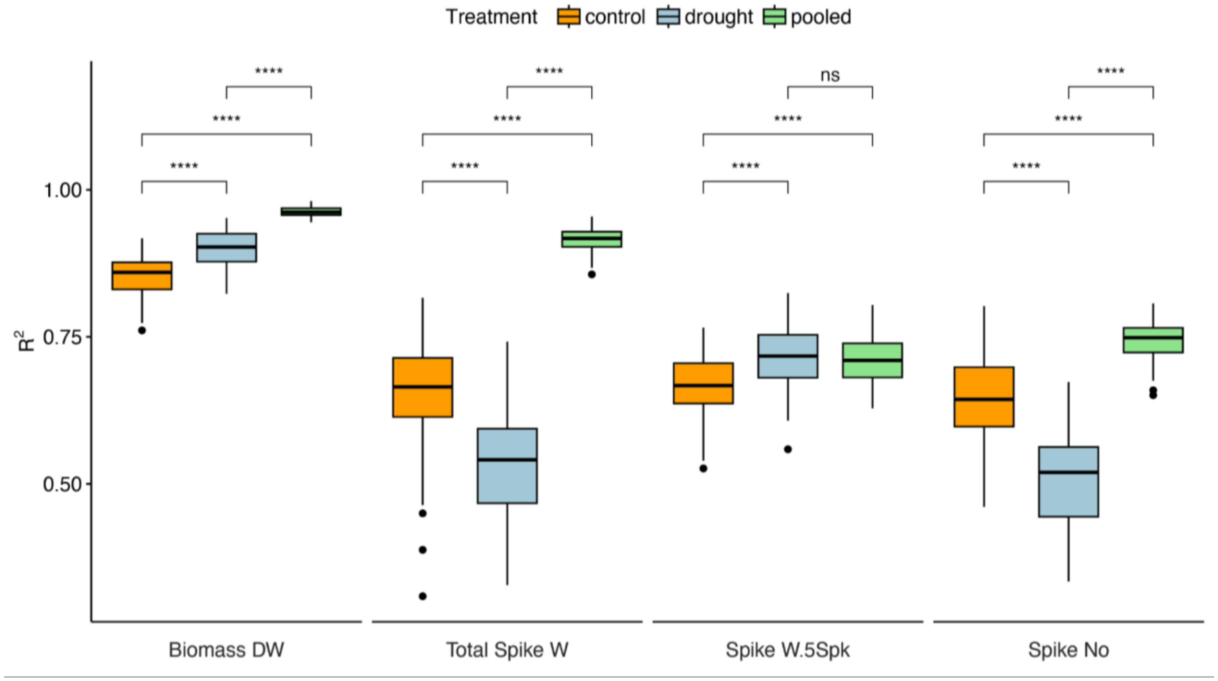

Supplementary Figure S7. Comparison between the different treatments using the LASSO model. Boxplots showing the accuracy (determined by R^2) among selected harvested traits using the LASSO model trained on a non-aggregated dataset. The significance level was determined as **** for P < 0.0001 and ns for non-significant differences between the models.

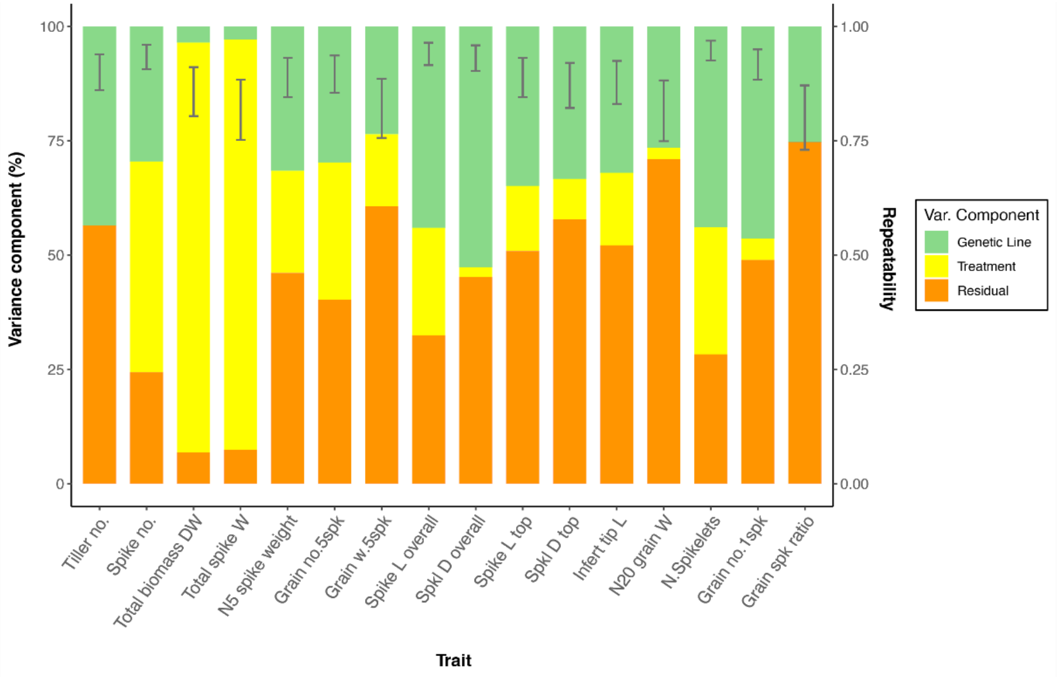

Supplementary Figure S8. Partitioning of variances and repeatability of the harvest-related traits. Repeatability on the secondary y-axis is represented as error bars.

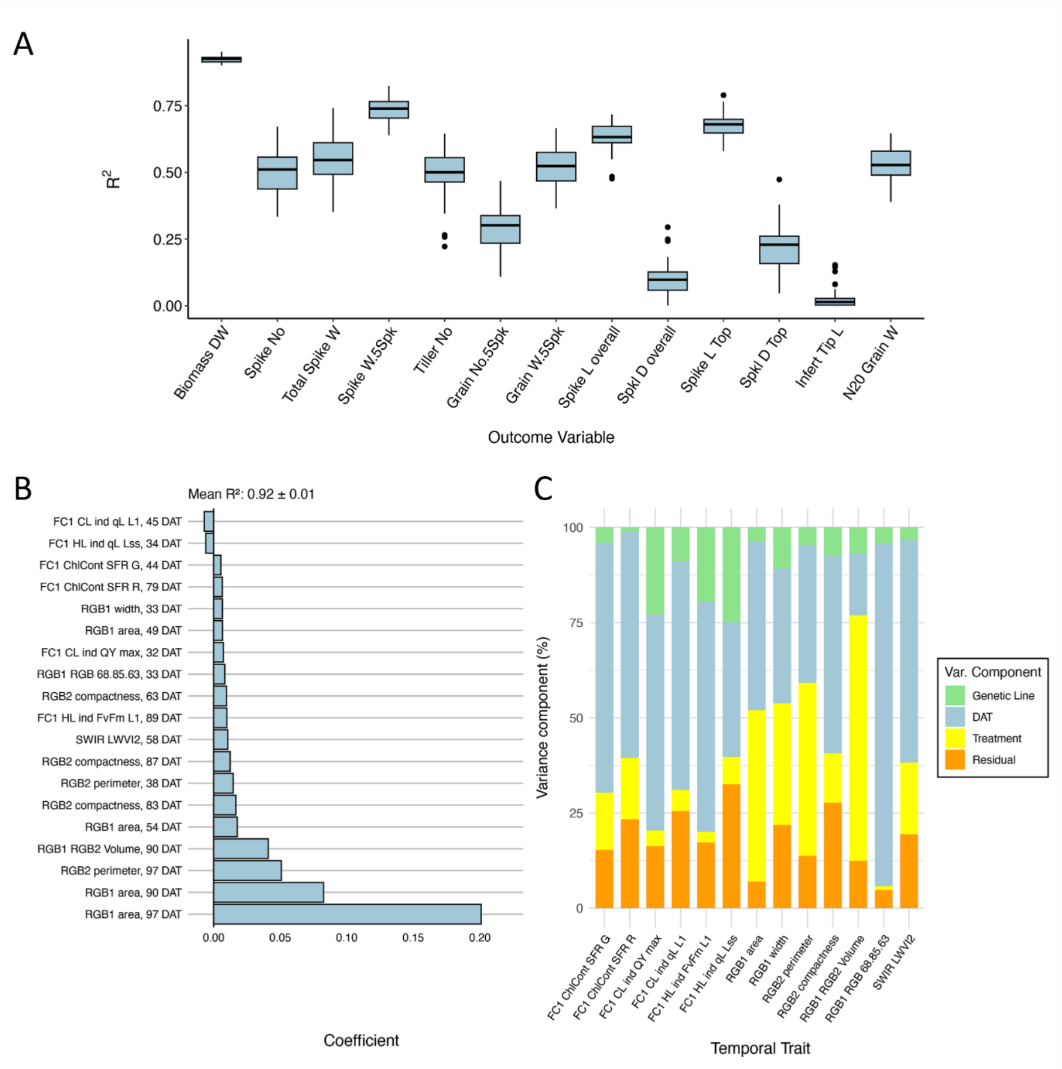

Supplementary Figure S9. Harvest trait prediction models on drought non-aggregated dataset. **(A)** Boxplots showing the accuracy (determined by R^2) among all the harvested traits using the LASSO model trained on drought non-aggregated dataset. **(B)** LASSO coefficients on the total biomass dry weight where coefficients with absolute values below 0.005 are not plotted. **(C)** Partitioning of variances of the temporal traits selected from LASSO model.

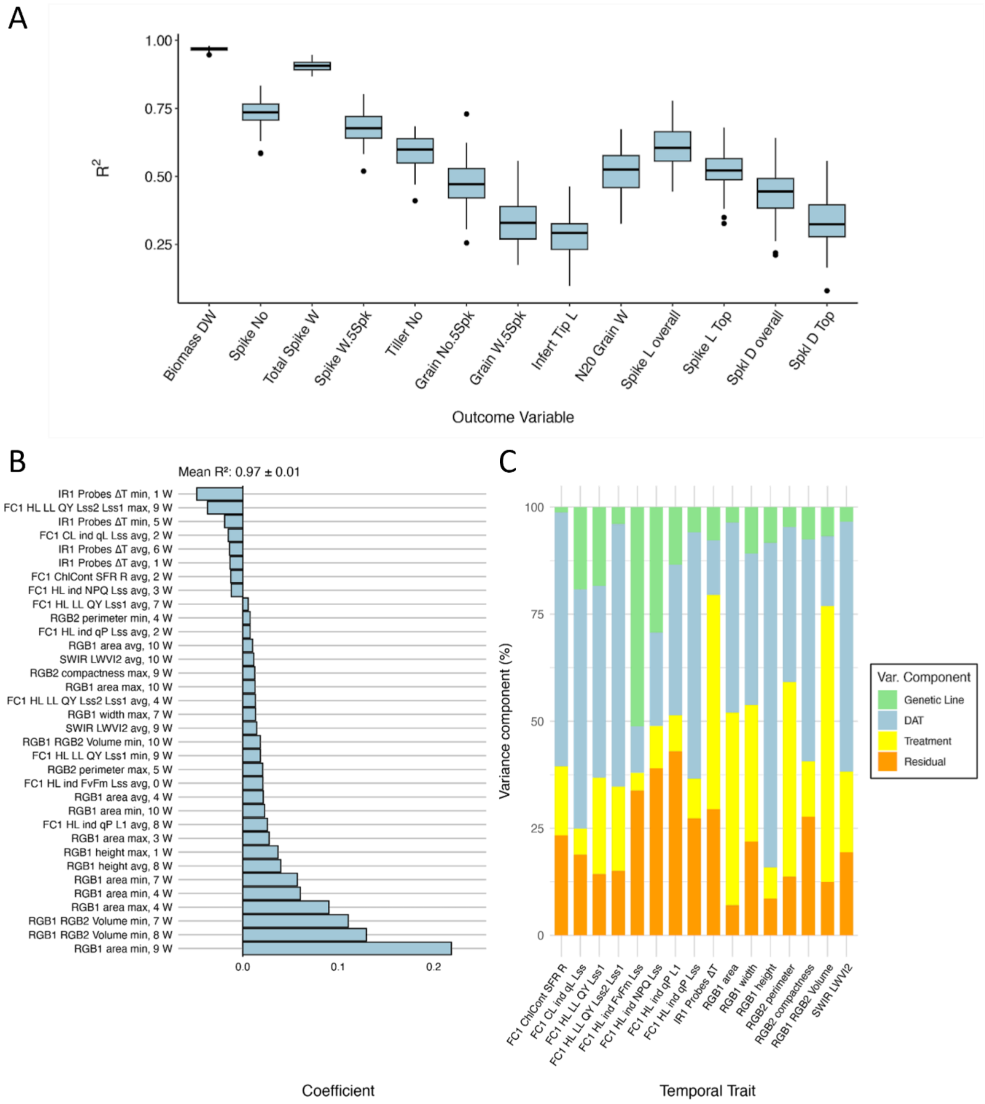

Supplementary Figure S10. Harvest-traits prediction models on an aggregated dataset. (A) Boxplots showing the accuracy (determined by R^2) among all the harvested traits using the LASSO model trained on pooled aggregated dataset. **(B)** LASSO coefficient on the total biomass dry weight. **(C)** Partitioning of variances of the temporal traits selected from LASSO model.

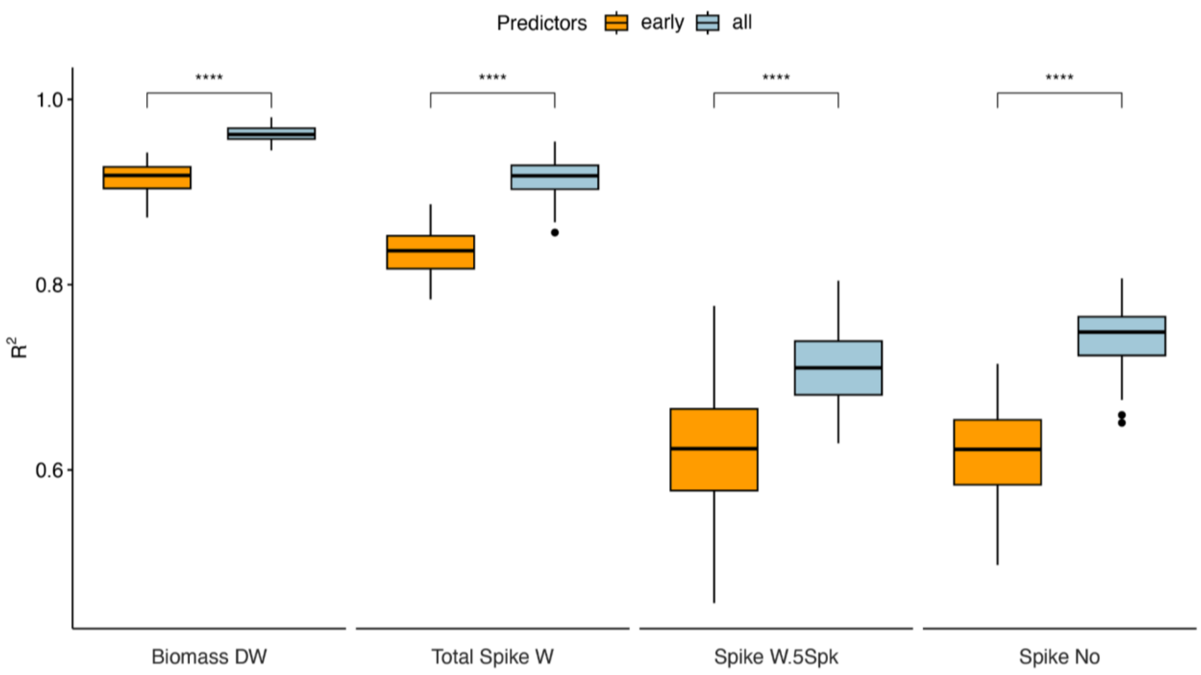

Supplementary Figure S11. Comparison between the different early and all time points using the LASSO model. Boxplots showing the accuracy (determined by R^2) among selected harvested traits using the LASSO model trained on pooled non-aggregated dataset. Early time points were selected until 51 days after transplant (DAT). The significance level was determined as **** for P < 0.0001 and ns for non-significant differences between the models.

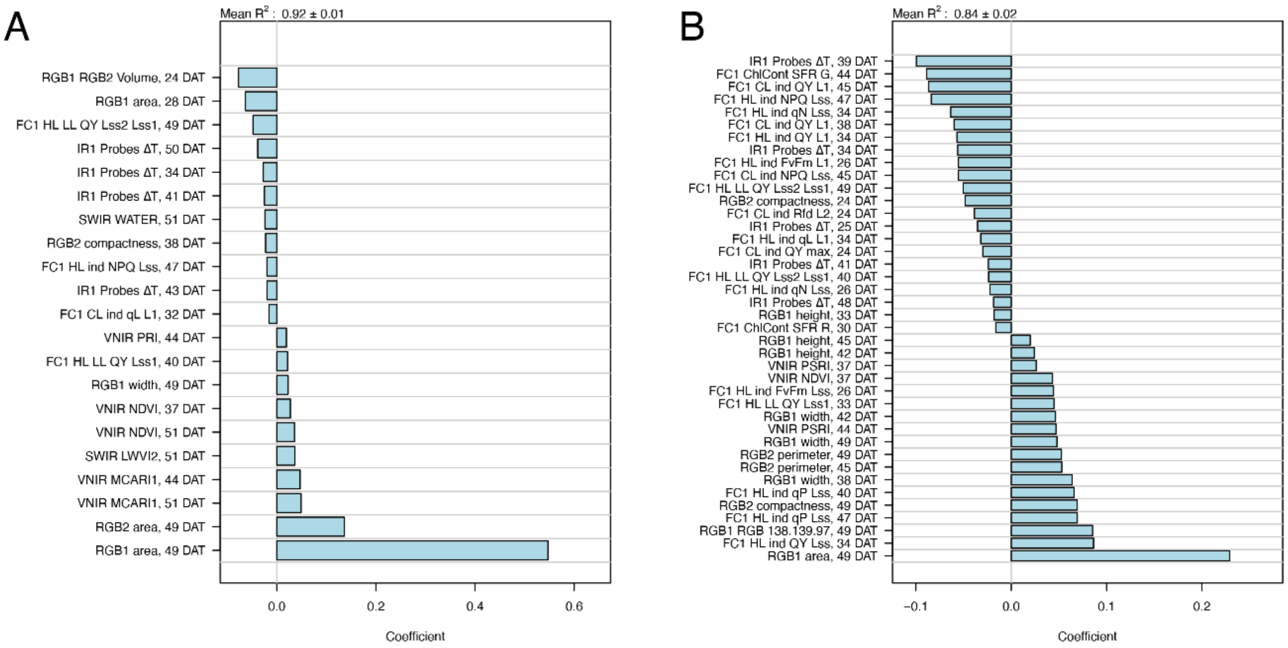

Supplementary Figure S12. LASSO coefficients of early time point TPP models for harvest traits. (A) Biomass dry weight and **(B)** total spike weight were selected. Only coefficients with absolute values above 0.015 are plotted.

SUPPLEMENTARY TABLES

Population	ID	Description	Alleles group	
**Elite cultivar**	L1	Barke		cultivated
**CMPP**	L2	B1K-29-13xNoga	Hs/Hs	wild
	L3	B1K-29-13xNoga	Hv/Hv	cultivated
	L4	B1K-09-07xNoga	Hs/Hs	wild
	L5	B1K-09-07xNoga	Hv/Hv	cultivated
**HEB-25**	L6	HEB-04-096xHEB-16-037	Hs/Hs	wild
	L7*	HEB-04-096xBarke	Hs/Hs	wild
	L8*	HEB-04-096xBarke	Hv/Hv	cultivated
**Elite cultivar**	L9*	Noga		cultivated

* Excluded from analyses that assumed genetic homogeneity (i.e., clustering of samples, drought tolerance ranking, and trait variance decomposition).

Supplementary Table S1. Genetic background of the plant material for the selected lines.

Sensor (PID)	Parameter	Unit	Description	Formula
FC	Fo	arbitrary unit (a.u.)	Minimum fluorescence in dark-adapted state	Measured
	Fm	a.u.	Maximum fluorescence in dark-adapted state	Measured
	Fv	a.u.	Variable fluorescence in dark-adapted state	Fm – Fo
	Fp	a.u.	Peak fluorescence during the initial phase of the Kautsky effect	Measured
	Fm_L1	a.u.	Maximum fluorescence under actinic light 1	Measured
	Fm_L2	a.u.	Maximum fluorescence under actinic light 2	Measured
	Fm_Lss	a.u.	Steady-state maximum fluorescence in light	Measured
	Ft_L1	a.u.	Instantaneous fluorescence under actinic light 1	Measured
	Ft_L2	a.u.	Instantaneous fluorescence under actinic light 2	Measured
	Ft_Lss	a.u.	Steady-state fluorescence in light	Measured
	Fo_L1	a.u.	Minimum fluorescence under actinic light 1	Measured
	Fo_L2	a.u.	Minimum fluorescence under actinic light 2	Measured
	Fo_Lss	a.u.	Steady-state minimum fluorescence in light	Fo/((Fv/Fm)+(Fo/Fm_Lss))
	Fv_L1	a.u.	Variable fluorescence under actinic light 1	Fm_L1 - Fo_L1
	Fv_L2	a.u.	Variable fluorescence under actinic light 2	Fm_L2 - Fo_L2
	Fq_L1	a.u.	Difference in fluorescence between Fm_L1 and Ft_L1 in light	Fm_L1 – Ft_L1
	Fq_L2	a.u.	Difference in fluorescence between Fm_L2 and Ft_L2 in light	Fm_L2 – Ft_L2
	Fq_Lss	a.u.	Fluorescence quenching capacity under actinic light	Fm_Lss – Ft_Lss
	QY_max	a.u.	Maximum quantum efficiency of PSII photochemistry in dark-adapted state	Fv/Fm
	Fv/Fm_L1	a.u.	PSII maximum efficiency of light adapted sample	Fv_L1/Fm_L1
	Fv/Fm_L2	a.u.	PSII maximum efficiency of light adapted sample	Fv_L2/Fm_L2
	Fv/Fm_Lss	a.u.	PSII maximum efficiency of light adapted sample in steady-state	Fv_Lss/Fm_Lss
	QY_ L1	a.u.	Instantaneous PSII quantum yield under actinic light 1	Fq_L1/Fm_L1
	QY_ L2	a.u.	Instantaneous PSII quantum yield under actinic light 2	Fq_L2/Fm_L2
	QY_ Lss	a.u.	Steady-state PSII quantum yield in light	Fq_Lss/ Fm_Lss
	NPQ_ L1	a.u.	Instantaneous non-photochemical quenching under actinic light 1	(Fm-Fm_L1)/ Fm_L1
	NPQ_ L2	a.u.	Instantaneous non-photochemical quenching under actinic light 2	(Fm-Fm_L2)/ Fm_L2

	NPQ _Lss	a.u.	Steady-state non-photochemical quenching	(Fm - Fm_Lss)/Fm_Lss
	qN_L1	a.u.	Coefficient of non-photochemical quenching under actinic light 1	(Fm-Fm_L1)/ (Fm- Fo_L1)
	qN_L2	a.u.	Coefficient of non-photochemical quenching under actinic light 2	(Fm-Fm_L2)/ (Fm- Fo_L2)
	qN_Lss	a.u.	Coefficient of non-photochemical quenching in steady state	(Fm-Fm_Lss)/ (Fm- Fo_Lss)
	qP_Ln1	a.u.	Coefficient of photochemical quenching under actinic light 1	Fq_L1/Fv_L1
	qP _Ln2	a.u.	Coefficient of photochemical quenching under actinic light 2	Fq_L2/Fv_L2
	qP_Lss	a.u.	Coefficient of photochemical quenching in steady-state	Fq_Lss/Fv_Lss
	qL_L1	a.u.	Fraction of PSII centers that are 'open' under actinic light 1	(Fq_L1/Fv_L1) *(Fo/Ft_L1)
	qL_L2	a.u.	Fraction of PSII centers that are 'open' under actinic light 2	(Fq_L2/Fv_L2) *(Fo/Ft_L2)
	qL_Lss	a.u.	Fraction of PSII centers that are 'open' in steady state	(Fq_Lss/Fv_Lss)* (Fo/Ft_Lss)
	Rfd_L1	a.u.	Instantaneous fluorescence decline ratio under actinic light 1	(Fp-Ft_L1) / Ft_L1
	Rfd_L2	a.u.	Instantaneous fluorescence decline ratio under actinic light 2	(Fp-Ft_L2) / Ft_L2
	Rfd Lss	a.u.	Fluorescence decline ratio in steady-state	(Fp - Ft_Lss) /Ft_Lss
	QY_Lss2/Lss1	a.u.	Ratio between steady-state QY measured under low (Lss2) and high (Lss1) light	QY_Lss_LL/QY_Lss_HL
	FRF_G	a.u.	Measurement of Far-Red Chlorophyll Fluorescence (FRF) at green light excitation	Measured
	RF_Gcorr	a.u.	Measurement of Red Chlorophyll Fluorescence (RF) at green light excitation	RF_G*3
	SFR_G	a.u.	Ratio of Red and Far-Red Chlorophyll Fluorescence at green light excitation	RF_G/FRF_Gcorr
	FRF_R	a.u.	Measurement of Far-Red Chlorophyll Fluorescence at red-orange light excitation	Measured
	RF_Rcorr	a.u.	Measurement of Red Chlorophyll Fluorescence at red-orange light excitation	RF_R*3
	SFR_R	a.u.	Ratio of Red and Far-Red Chlorophyll Fluorescence at red-orange light excitation	RF_R/FRF_Rcorr
**SWIR**	WATER1	a.u.	Water Content Index trait based on hyperspectral short wave Infrared (SWIR) top view	R1440/R960
	LWVI2	a.u.	Leaf Water Vegetation Index2	(R1094 - R1205) / (R1094 + R1205)

VNIR	PRI	a.u.	Photochemical Reflectance Index trait based on hyperspectral visible near-infrared (VNIR) top view	(R531 - R570) / (R531 + R570)
	NDVI	a.u.	Normalized Difference Vegetation Index trait based on hyperspectral VNIR top view	(R800 - R670) / (R800 + R670)
	PSRI	a.u.	Plant Senescence Reflectance Index trait based on hyperspectral VNIR top view	(R680 - R500) / R750
	OSAVI	a.u.	Optimized Soil-Adjusted Vegetation Index trait based on hyperspectral VNIR top view	(1 + 0.16) * (R800 - R670) / (R800 + R670 + 0.16)
	SIPI	a.u.	Structure Insensitive Pigment Index trait based on hyperspectral VNIR top view	(R790 - R450) / (R790 + R650)
	MCARI1	a.u.	Modified Chlorophyll Absorption in Reflectance Index trait based on hyperspectral VNIR top view	1.2 * (2.5 * (R800 - R670) - 1.3 * (R800 - R550))
**IR1**	Temp-avg	°C	Average temperature of the entire plant surface	
**IR1-Probes**	canopy temperature depression (ΔT)	°C	Difference between leaf temperature and air temperature in the imaging unit, known as delta Temperature	Temp-avg - Temp-air
**RGB1**	Area	mm^2	Total area covered with plant	
	Height	mm	Height of bounding box enveloping plant	
	Width	mm	Width of bounding box enveloping plant	
**RGB2**	Area	mm^2	Total area covered with plant	
	Perimeter	mm	Length of the plant perimeter, usually used for further computations	
	Compactness	a.u.	Ratio between area and surface of convex hull enveloping particular plant	Area / Convex_Hull_Area
	SOL	a.u.	Solidity of the plant, Geometry trait based on visible-light top view	Ratio between square of leaf lengths (sum of distances from plant center to the end of a leaf blade) and area
**RGB1/RGB2**	Volume	mm^3	Geometry trait based on visible-light combined view	Volume estimation according to this calculation: sqrt((SideArea)^2* (TopArea)
	Plant Color Segmentation	a.u.	The software calculates the proportion of the plant which falls into color groups pre-selected by the user For example, RGB (32,37,31) RGB (110,110,89), etc.	
**SC**	weight	g	Weight (absolute weight trait based on weighing of the entire pot)	Absolute weight of the pot
	weight after watering	g	Weight after watering (absolute weight trait based on weighing of the entire pot after watering of the pot)	Absolute weight of the pot after completion of watering

	water consumption	g/day	Absolute weight trait based on weighing of the entire pot over time	Water consumption estimation according to this calculation: (Weight after watering (Time n) - Weight before watering (Time n+1))
	Cumulative water	g	The sum of water consumed daily between two consecutive days	The sum of water consumed daily between two consecutive days, Time point 0+1 then Time point 1+2, etc.
**Harvest**	Tiller No		Tiller number per plant	
	Spike No		Spike number per plant	
	Biomass DW	g	Total Biomass dry weight per plant	
	Total Spike W	g	Total Spike weight per plant	
	Spike W.5Spk	g	Spike weight per 5 spikes	
	Grain No.5Spk		Grain number per 5 spikes	
	Grain W.5Spk	g	Grain weight per 5 spikes	
	Spike L overall	cm	Overall spike length per spike	
	Spkl D overall	cm	Spikelet density to overall spike length	Number of Spikes / Overall Spike Length (cm)
	Spike L Top	cm	Spike length to base of top grain per spike	
	Spkl D Top	cm	Spikelet density to the base of top spike	Spike Density = Number of Spikes / Spike Length to Base of Top Spike (cm)
	Infert Tip L	cm	Infertile tip length per spike	
	N20 Grain W	g	Grain weight of 20 grains per plant	
	Spkl No		Spikelet number accessed from X-ray image of 5 spikes	
	Grain No.1Spk		Grain number per spike accessed from X-ray image of 5 spikes	
	Grain Spk ratio		Average number of grains per spike to assess fertility per spike	

Supplementary Table S2. List of temporal traits description from multiple imaging sensors and harvest-related traits.

Group	Missing Samples	OOB Error
L2, Control	1	0.0348
L5, Control	1	0.0660
L7, Control	2	0.0534
L7, Drought	4	0.0544
L8, Drought	1	0.0506

Supplementary Table S3. Out of bag (OOB) error of imputed missing values in harvest traits. In all samples, missing values were only in the total spike weight trait, except in L8, where total biomass was also missing. OOB error is calculated as normalized root mean square error (NRMSE) based on predictions for observed values that were left out of the training bootstrap samples (i.e., out-of-bag) when fitting the random forest.

Sensor type	Outlier Rate	Re-imputation OOB Error mean
FC, Night protocol using conditional light	0.0092	0.0194 ± 0.0322
FC, Night protocol using high light	0.0112	0.0209 ± 0.0337
FC, Morning protocol for chlorophyll content	0.0090	0.0563 ± 0.0867
FC, Morning protocol for QY Lss	0.0155	0.0330 ± 0.0403
Thermal IR	0.01	0.0166 ± 0.0114
RGB	0.0057	0.0168 ± 0.0485
RGB color segmentation	0.0147	0.1481 ± 0.2816
HS, SWIR	0.0073	0.0336 ± 0.0379
HS, VNIR	0.0075	0.0184 ± 0.0401
Harvest	0.0084	0.0315 ± 0.0499

Supplementary Table S4. Outlier rate and re-imputation out of bag (OOB) error for each sensor type, including different protocols as described in Figure 1. Outliers were detected for each trait among all replicates within a genetic line within a treatment.

Sensor type	NN rate separated	NN rate corrected separated	NN rate pooled	NN rate corrected pooled
FC, Night protocol using conditional light	0.0818	0.0389	0.0475	0.0278
FC, Night protocol using high light	0.0693	0.0302	0.0455	0.0236
FC, Morning protocol for chlorophyll content	0.1528	0.0250	0.0611	0.0167
FC, Morning protocol for QY Lss	0.1240	0.0600	0.0773	0.0693
Thermal IR	0.1370	0.1304	0.0348	0.0130
RGB	0.0434	0.0202	0.0694	0.0480
RGB color segmentation	0.0620	0.0480	0.0640	0.0520
HS, SWIR	0.0150	0.0100	0.0350	0.0100
HS, VNIR	0.0370	0.0290	0.0540	0.0380
Harvest	0.0577	0.0231	0.0846	0.0462

Supplementary Table S5. Rates of non-normality (NN) for each sensor type, including different protocols as described in Figure 1. Non-normality was tested for each trait within each data group, either separated by treatment or pooled across treatments. Groups identified as non-normal were corrected using a Box–Cox transformation, and NN rates were recalculated post-correction.

DAT	WP	Developmental stage	Classification
24-29	0	Tillering	Early stage
30-36	1		
37-43	2	Stem elongation	
44-49	3		
50-57	4	Booting	Late stage
58-64	5	Flowering	
65-71	6		
72-78	7	Grain ripening and maturity	
79-85	8		
86-92	9		
93-98	10		

Supplementary Table S6. Mapping between the daily time point counting from days after transfer of seedlings to light (DAT) for the non-aggregated dataset and the corresponding weekly phases (WP) for the aggregated dataset. The developmental stage was marked when most of the replicates among all the genetic lines reached a certain stage. Early stage refers to the vegetative and early reproductive phases, during which plants were exposed to 3 weeks of drought stress. Late stage refers to the late reproductive phase, when the flag leaf was fully expanded, and plants were exposed to a longer duration of drought stress.

Response Trait	Training Group	R^2 Control	R^2 Drought	R^2 Pooled
Biomass DW	Control	0.935	0.876	0.894
Biomass DW	Drought	0.793	0.953	0.886
Biomass DW	Pooled	0.893	0.945	0.985
Spike No	Control	0.9	0.174	0.668
Spike No	Drought	0.272	0.822	0.539
Spike No	Pooled	0.852	0.821	0.898
Total Spike W	Control	0.958	0.483	0.849
Total Spike W	Drought	0.618	0.926	0.925
Total Spike W	Pooled	0.887	0.856	0.969
Spike W.5Spk	Control	0.785	0.649	0.654
Spike W.5Spk	Drought	0.589	0.816	0.587
Spike W.5Spk	Pooled	0.783	0.834	0.828

Supplementary Table S7. Performance of final LASSO models trained on the full predictor set using the optimal parameter (λ). Accuracies much higher than those found in the internal CV procedure during training suggest overfitting. "Training Group" refers to the data group used to train the model, while the R^2 values show the accuracy when the model is applied to the data group in question.
